# Dual-Transcriptomic, Microscopic, and Biocontrol Analyses of the Interaction Between the Bioeffector *Pythium oligandrum* and the Pythium Soft-Rot of Ginger Pathogen *Pythium myriotylum*

**DOI:** 10.3389/fmicb.2021.765872

**Published:** 2021-11-16

**Authors:** Paul Daly, Siqiao Chen, Taiqiang Xue, Jingjing Li, Taha Majid Mahmood Sheikh, Qimeng Zhang, Xuehai Wang, Jinfeng Zhang, David A. Fitzpatrick, Jamie McGowan, Xiujuan Shi, Sheng Deng, Min Jiu, Dongmei Zhou, Irina S. Druzhinina, Lihui Wei

**Affiliations:** ^1^Key Lab of Food Quality and Safety of Jiangsu Province—State Key Laboratory Breeding Base, Institute of Plant Protection, Jiangsu Academy of Agricultural Sciences, Nanjing, China; ^2^Fungal Genomics Laboratory (FungiG), Jiangsu Provincial Key Lab of Organic Solid Waste Utilization, Nanjing Agricultural University, Nanjing, China; ^3^College of Food and Bioengineering, Henan University of Science and Technology, Luoyang, China; ^4^Jinan Academy of Agricultural Sciences, Jinan, China; ^5^Genome Evolution Laboratory, Maynooth University, Maynooth, Ireland; ^6^School of Environment and Safety Engineering, Jiangsu University, Zhenjiang, China

**Keywords:** *Pythium oligandrum*, bioeffector, dual RNAseq, antagonism, ginger, *Pythium myriotylum*

## Abstract

Biological control is a promising approach to suppress diseases caused by *Pythium* spp. such as Pythium soft rot of ginger caused by *P. myriotylum*. Unusually for a single genus, it also includes species that can antagonize *Pythium* plant pathogens, such as *Pythium oligandrum*. We investigated if a new isolate of *P. oligandrum* could antagonize *P. myriotylum*, what changes occurred in gene expression when *P. oligandrum* (antagonist) and *P. myriotylum* (host) interacted, and whether *P. oligandrum* could control soft-rot of ginger caused by *P. myriotylum*. An isolate of *P. oligandrum*, GAQ1, recovered from soil could antagonize *P. myriotylum* in a plate-based confrontation assay whereby *P. myriotylum* became non-viable. The loss of viability of *P. myriotylum* coupled with how *P. oligandrum* hyphae could coil around and penetrate the hyphae of *P. myriotylum*, indicated a predatory interaction. We investigated the transcriptional responses of *P. myriotylum* and *P. oligandrum* using dual-RNAseq at a stage in the confrontation where similar levels of total transcripts were measured from each species. As part of the transcriptional response of *P. myriotylum* to the presence of *P. oligandrum*, genes including a subset of putative Kazal-type protease inhibitors were strongly upregulated along with cellulases, elicitin-like proteins and genes involved in the repair of DNA double-strand breaks. In *P. oligandrum*, proteases, cellulases, and peroxidases featured prominently in the upregulated genes. The upregulation along with constitutive expression of *P. oligandrum* proteases appeared to be responded to by the upregulation of putative protease inhibitors from *P. myriotylum*, suggesting a *P. myriotylum* defensive strategy. Notwithstanding this *P. myriotylum* defensive strategy, *P. oligandrum* had a strong disease control effect on soft-rot of ginger caused by *P. myriotylum*. The newly isolated strain of *P. oligandrum* is a promising biocontrol agent for suppressing the soft-rot of ginger. The dual-RNAseq approach highlights responses of *P. myriotylum* that suggests features of a defensive strategy, and are perhaps another factor that may contribute to the variable success and durability of biological attempts to control diseases caused by *Pythium* spp.

## Introduction

Biological control is a promising strategy to suppress diseases in an environmentally friendly manner whereby microorganisms, the biocontrol agents (BCAs), can antagonize a plant pathogen and prime plant defenses (Köhl et al., 2019). The application of biocontrol strategies in the field is often hampered by a lack of robustness where the previously observed disease protection effect is not maintained. One area needing further investigation is molecular aspects of the interaction between a plant pathogen and a biocontrol agent or in terms appropriate to their ecological interactions—the host or prey and the microbial antagonist.

One prominent biocontrol species is the oomycete *Pythium oligandrum*, which has several known mechanisms of antagonism contributing to its ability to control plant diseases as well as plant-mediated mechanisms. Recently, the review of Thambugala et al. (2020) summarized plant diseases caused by fungi or oomycetes that could be controlled by *P. oligandrum*. In particular, with regard to diseases caused by oomycetes, most of the diseases where *P. oligandrum* has a control effect are caused by *P. ultimum*, which causes diseases on a broad range of crop species (Thambugala et al., 2020). E.g., *P. oligandrum* has been reported to control pre- and post-emergence damping-off of wheat caused by *P. ultimum* (Abdelzaher et al., 1997). To our knowledge, there are no reports in the literature of antagonism of *P. oligandrum* toward *P. myriotylum* or to the control of diseases caused by *P. myriotylum*. An unpublished report showed that an isolate of *P. oligandrum* was antagonistic toward *P. myriotylum* in plate confrontation assays, but there was no control effect on Pythium soft-rot (PSR) of ginger caused by *Pythium myriotylum* (Le, 2016).

*P. myriotylum* is a broad host range pathogen that can infect a range of monocot and dicot plant species (van der Plaats-Niterink, 1981). One of these monocot hosts is ginger (*Zingiber officinale*) and recently, it was shown that *P. myriotylum* can be the most frequently found oomycete species from infected ginger rhizomes showing symptoms of PSR in the major ginger growing regions in China (Daly et al., 2021). Ginger is a spice and an important crop (De-Guzman and Siemonsma, 1999), with global production in 2019 estimated at approximately four million metric tons (FAOSTAT, 2019). Control strategies for PSR of ginger were comprehensively reviewed by Le et al. (2014) and are primarily based on chemical pesticide treatments. There is also potential for biological control methods such as a report of using a hypocrealean fungus *Trichoderma harzianum* to control PSR of ginger (Singh, 2011), but as reviewed by Le et al. (2014), the efficacies of the biological treatments appear lower in field conditions. In light of this, there is a need to identify novel biological control agents to control PSR of ginger.

Recently, the genome of the *P. oligandrum* ATCC 38472 strain was sequenced by long-read sequencing technology (Faure et al., 2020), and previously the *P. oligandrum* strains Po37 (Berger et al., 2016) and CBS 530.74 (Kushwaha et al., 2017) were sequenced by short-read sequencing technology. Note that the ATCC 38472 strain of *P. oligandrum* is used in the commercial oospore-containing product Polyversum^®^. In a comparative genomics analysis of *P. oligandrum* with other oomycetes, there was an expansion of carbohydrate-active enzyme (CAZy) families (Lombard et al., 2014) involved in the degradation of cellulose (Liang et al., 2020), perhaps related to the ability of *P. oligandrum* to degrade the cellulose in the cell wall of an oomycete host or prey. *P. oligandrum* is often referred to as a mycoparasite as its antagonism includes the coiling of *P. oligandrum* hyphae around hyphae of its host (Berry et al., 1993). *P. oligandrum* possesses an arsenal of hydrolytic enzymes such as cellulases and proteases that are important for antagonism toward particular hosts. The CAZyme content of *P. oligandrum* was described recently by Liang et al. (2020). The production of antimicrobial compounds by *P. oligandrum* also contributes to the antagonism (Benhamou et al., 1999). Interestingly, *P. oligandrum* can enter and colonize the root tissue before it rapidly degrades or degenerates itself (Benhamou et al., 2012). Presumably, this rapid degeneration of *P. oligandrum* can limit the damage that its cellulases can cause to the plant cell wall, of which cellulose is a major component. *P. oligandrum* appears to have a priming role in stimulating plant defense responses by microbe-associated molecular pattern (MAMP) production such as the elicitin-like protein oligandrin (Benhamou et al., 2001). Recently, type II NLP toxin proteins from *P. oligandrum* were found to suppress *Phytophthora capsici* infection (Yang et al., 2021). Expression of putative effectors was found when *P. oligandrum* interacted with heat-killed *Phytophthora infestans* mycelia (Horner et al., 2012), and these could potentially antagonize the host or mediate plant defense responses.

Dual transcriptomics simultaneously analyses the transcriptomes of two or more interacting organisms and is frequently used in host-pathogen interaction (Westermann et al., 2017). This can uncover expression differences in interacting organisms such as the upregulation of genes related to antagonism when three saprobic ascomycete fungi were cultured together (Daly et al., 2017). Dual-RNAseq was used to uncover the transcriptional responses of the biocontrol rhizobacterium *Lysobacter capsici* and the pathogen *Phytophthora infestans* (Tomada et al., 2017). In this interaction, *L. capsici* genes involved in host colonization and degradation, and detoxification were upregulated while in contrast the host *P. infestans* was not considered to have mounted a major defensive or counter-attacking response but a transcriptomic response perhaps related to cell death (Tomada et al., 2017).

Here we investigated if a new isolate of *P. oligandrum* could antagonize *P. myriotylum* and used dual-RNAseq to measure the changes in gene expression that occurred when the two species confronted each other, alongside investigating if *P. oligandrum* had a control effect on the disease caused by *P. myriotylum*.

## Materials and Methods

### *Pythium* Strains and Routine Culturing

*P. oligandrum* GAQ1 CGMCC No. 17470 (Wei et al., 2019) was isolated from soil from a field where infected ginger was growing in Laiwu district, Jinan City, Shandong Province, China. The molecular marker sequences of the GAQ1 isolate (MK774755.1, MZ891585.1, MZ891586.1 and MZ869812.1) have been deposited in GenBank. The *P. myriotylum* SWQ7 CGMCC No. 21459 isolate was described previously by Daly et al. (2021). The *Pythium* species were routinely cultured on 10% V8 juice agar at 25°C and maintained on V8 juice agar slants at 12°C.

### Identification of *P. oligandrum* GAQ1

The CTAB protocol of Doyle (1990) was used to extract DNA from mycelial mats growing on V8 juice agar. The ITS region including the 5.8S rRNA subunit was amplified using the two universal primers ITS1 and ITS4 (White et al., 1990). The *CoxI* gene was amplified using OomCoxILevup and OomCoxI-Levlo primers (Robideau et al., 2011), the *CoxII* gene using FM66 and F58 primers (Martin and Tooley, 2003), and the β-tubulin gene using TUBUF2 and TUBUR1 primers (Kroon et al., 2004). PCR reactions were performed using a proof-reading polymerase with standard reaction and cycling conditions. All PCR products were run on an agarose gel to confirm the presence of an amplified product. The sequencing of the PCR products, BLAST searches and phylogenetic analysis were performed as described previously (Daly et al., 2021).

### Visual Analysis of *P. oligandrum* and *P. myriotylum* Interaction

From fresh pre-culture plates on V8 juice media, an agar plug from the hyphal front was used to inoculate onto opposite ends of V8 agar on 9 cm plates and incubated at 25°C. For the macroscopic observation of the colonies, 20 mL of V8 agar was added to the plate, and for the microscopic visualization, 4.5 ml of V8 agar was added to the plates to provide a thinner layer. 5 mL of a 0.01% w/v trypan blue solution was used to cover the mycelium and agar surface. Trypan blue is a type of vitality stain, and it stains non-viable tissues with a blue color. The plates were gently rotated to ensure that the dye and mycelium were in full contact, and then incubated without shaking at room temperature for 3 min. Then the staining solution was decanted, and any remaining dye was pipetted off. The stained mycelial cultures were washed twice with 5 ml of water by incubating for 5 min without shaking. The staining was done after contact of the hyphal fronts and at various stages afterward. Microscopic visualization was performed using a brightfield microscope (NE900, Nexcope). At least three replicate cultures were used for the visualization work. A cryo-scanning electron microscope (cryo-SEM) (Quorum PP3010T system integrated onto a Hitachi SU8010 FE-SEM) was also used to visualize the interaction between *P. oligandrum* and *P. myriotylum*.

### Dual-RNAseq Transcriptomics Experiment

Pre-cultures were grown on 10% V8 juice agar at 25°C. From the edge of the colony of *P. myriotylum* SWQ7 or *P. oligandrum* GAQ1, 0.5 cm mycelial plugs were transferred to 10% V8 juice agar covered with a 14.2 cm diameter polycarbonate membrane with a 0.1 μm pore size (GVS filter technology, Cat. no. 1215304) in a 14 cm Petri dish. The mycelial plugs were placed equidistant from each other and the edge of the Petri dish. The Petri dishes were inoculated with either two plugs of the same species (self-confrontation) or one plug from each species (mixed species confrontation). The cultures were incubated at 25°C in the dark, and mycelia from the confrontation zone was sampled as shown by the dotted-lines (Supplementary Figure 1). The mycelium was flash-frozen in liquid nitrogen before being ground using a Tissue Lyser. The sample was mixed with 1 mL of Trizol before adding 200 μl chloroform: isoamyl alcohol (24:1). The RNA was precipitated by mixing 0.8 volume of isopropanol with the aqueous phase from the previous step and the resulting pellet was washed once with 70% EtOH before resuspension in DEPC-treated water. The RNA was then cleaned up using a Tiangen Plant RNA extraction kit which included an on-column DNase treatment. RNA integrity was visualized using an agarose gel and a Bioanalyzer, and RNA quantity and purity using a NanoDrop spectrophotometer.

### RNA Sequencing and Data Analysis

A paired library 2 × 150 bp was prepared using the TruSeq Stranded mRNA LT Sample Prep Kit (Illumina) and sequenced using the MGISEQ-2000 platform (MGI Tech Co. Ltd). The raw reads were processed using Trimmomatic (Bolger et al., 2014). The reads containing poly-N and the low-quality reads were removed to obtain the clean reads. Downstream analysis of the cleaned reads was performed using the following software tools in UseGalaxy.eu (Afgan et al., 2018). For all samples, reads that passed the filters were mapped using HISAT2 Galaxy Version 2.1.0 (Kim et al., 2019) onto a composite reference genome that consisted of the genomes of both species. The composite genome was made by concatenating into a single file those Fasta files made of the sequences for the scaffolds from *P. oligandrum* ATCC 38472 GCA_005966545.1_ASM596654v1_genomic.fasta (Faure et al., 2020) and *P. myriotylum* SWQ7 JAAS_PmSWQ7_1_0_assembly.fsa (PRJNA692555) (manuscript in preparation). The use of this composite genome facilitated the exclusion from subsequent analysis of reads that were not unique to a particular species as these reads would not be uniquely mapped in the composite genome. Uniquely mapped read counts (at the MAPQ10 threshold) for each gene were calculated using HTSeq-count Galaxy version 0.9.1^1^ (Anders et al., 2015). Read counts were calculated for each species separately using a genome annotation file for each species that contained the known gene exon coordinates for the genes for that species. The genome annotation file used for *P. oligandrum* was GCA_005966545.1_ASM596654v1_genomic.gff (Faure et al., 2020). Principal component analysis (PCA) indicated if the biological replicates were sufficiently similar for subsequent statistical analysis. The read counts of genes values for each species were subsequently used for statistical analysis using DESeq2 Galaxy Version 2.11.40.6 (Love et al., 2014). Note that the Cook’s distance cut-off was not used for outlier filtering. The criteria for a differentially expressed (DE) gene were an FPKM > 10 in one condition, DESeq p*_*adj*_* value < 0.05 and a DESeq log2 FC > 1 or < −1. FPKM normalized read counts were defined originally by Mortazavi et al. (2008). In our study, an FPKM value for a gene is the number of uniquely mapped (to the composite genome) fragments per kilobase of gene model per million uniquely mapped (to the composite genome) fragments onto gene models from a species. The RNA-seq reads from this project were submitted to the GEO database (GEO accession GSE179387).

### Gene Annotations of *P. myriotylum* and *P. oligandrum* Genes

For the *P. oligandrum* genes, the annotations of Faure et al. (2020) for the ATCC 38472 strain were used. The *P. myriotylum* genes were functionally annotated primarily using InterProScan (Jones et al., 2014), and the CAZymes were annotated using dbCAN2 (Zhang et al., 2018; manuscript in preparation). The annotations for the *P. oligandrum* and *P. myriotylum* genes are listed in Supplementary Table 1.

### qPCR Validation of Expression Differences in Dual-RNAseq

The qPCR validation was performed using RNA samples extracted from triplicate repeat cultures using the same experimental conditions and extraction method as described for the RNAseq experiment. cDNA was synthesized using the EasyScript one step gDNA removal and cDNA synthesis kit (TransGen Biotech) using random primers and 2.5 μg of total RNA according to the manufacturer’s instructions. The qPCR was performed with a LightCycler 96 Instrument (Roche Life Sciences) using the ChamQ SYBR qPCR Master Mix (VAZyme). The cycling conditions were 95°C for 3 min, followed by 40 cycles of 95°C for 10 s and 60°C for 30 s. A melt curve analysis step was included to confirm the specificity of the primer pairs used. A standard curve of fivefold serial dilutions of the gDNA from *P. myriotylum* SWQ7 and *P. oligandrum* GAQ1 was used to determine the primer efficiency. The data was analyzed using the LightCycler 96 software. To quantify the abundance of the target in the cDNA samples, the relative standard curve method was used, and the expression levels of the gene of interest were normalized to two reference genes. A tubulin gene (*Pm_g6466.t1*) and a gapdh gene (*Pm_17033.t1*) were used for *P. myriotylum*, and a succinate dehydrogenase gene (*Po_g2472.t1*) and a g6pdh gene (*Po_g6912.t1*) were used for *P. oligandrum*. A pair of primers that amplified from gDNA was used to confirm the efficiency of the gDNA removal treatments for the gDNA from each species. Primers were designed using Primer-BLAST software (Ye et al., 2012) at NCBI and the primers used are listed in Supplementary Table 2. The primer efficiency for the primer pairs used was at least 86% and generally > 90%, as detailed in Supplementary Table 2. Melt curve and agarose gel analysis were used to confirm the specificity of the primer pairs along with sequencing of the PCR products. A confirmation that the primers designed for *P. myriotylum* were unable to amplify from *P. oligandrum* and *vice-versa* was also performed.

### Ginger Pot-Trial With *P. myriotylum* and *P. oligandrum* to Determine Biocontrol Effect

*P. myriotylum* was cultured on 10% V8 juice agar for 1–2 day, and then twice-autoclaved wheat seeds (which had previously been soaked overnight in water) were plated onto the mycelial mat of *P. myriotylum* and incubated for a further 7 d or until the seeds were fully covered with mycelia. The wheat seeds from the *P. myriotylum* cultures (after scraping away the excess mycelium on the outside of the seed) were used to inoculate the vermiculite near the roots at a depth of 2–3 cm. *P. oligandrum* was inoculated using four mycelia-covered balls from liquid culture. The use of the wheat seed inoculum method for *P. myriotylum* was based on the sorghum seed inoculum method of Le et al. (2016). To produce the *P. oligandrum* inoculum, *P. oligandrum* was incubated on 10% V8 juice agar for 1 day at 25°C, and then 20 agar plugs of a diameter of 0.5 cm were used to inoculate 50 mL of 10% V8 juice medium in a 150 mL flask, then incubated for 3 d at 25°C and 100 rpm. A mycelium-based inoculum was used for *P. oligandrum* because oospore production has not been optimized to achieve a yield that can practically be used as an inoculum. Ginger plants (“Laiwu” variety) were derived from tissue culture and transplanted to autoclaved vermiculite in 100 mL pots with an inverted Petri dish lid placed underneath each pot (to hold the water and prevent cross-contamination of the pathogen), and grown in 16 h light and 8 h dark cycles at 25°C in a growth chamber. Periodically, the ginger plants were given a water-soluble fertilizer (NPK 20-20-20 + TE). Eighteen ginger plants (grown in individual pots) were used for each of the six test or control conditions (Table 1). After 7 days from deflasking of the ginger seedlings, the experiment was initiated by inoculating three conditions with *P.* oligandrum, and two of these *P. oligandrum*-inoculated conditions were later inoculated with *P. myriotylum* at either 7 or 14 days while the third *P. oligandrum*-inoculated condition was not inoculated with *P. myriotylum*. Two of the other conditions were inoculated only with *P. myriotylum* at either 7 or 14 days, and the last condition was not inoculated with either of the two species.

**TABLE 1:** Experimental design for ginger pot-trial with *P. myriotylum* and *P. oligandrum* to determine biocontrol effect.

	Controls		Test of 7 days between bioeffector and pathogen inoculations		Test of 14 days between bioeffector and pathogen inoculations	
Condition =	*P. oligandrum* alone inoculation	No inoculation	*P. oligandrum* and *P. myriotylum* inoculations	*P. myriotylum* inoculation	*P. oligandrum* and *P. myriotylum* inoculations	*P. myriotylum* inoculation

n (plants) =	18	18	18	18	18	18
Day 0	*P. oligandrum* inoculation	—	*P. oligandrum* inoculation	—	*P. oligandrum* inoculation	—
Day 1	—	—	—	—	—	—
Day 2	—	—	—	—	—	—
Day 3	—	—	—	—	—	—
Day 4	—	—	—	—	—	—
Day 5	—	—	—	—	—	—
Day 6	—	—	—	—	—	—
Day 7	—	—	*P. myriotylum* inoculations		—	—
Day 8	—	—	—	—	—	—
Day 9	—	—	—	—	—	—
Day 10	—	—	—	—	—	—
Day 11	—	—	—	—	—	—
Day 12	—	—	—	—	—	—
Day 13	—	—	—	—	—	—
Day 14	—	—	—	—	*P. myriotylum* inoculations	

Two different *P. myriotylum* inoculation time-points were used to show the biocontrol effect after different time periods between pathogen and bioeffector inoculation, and from ginger plants of different ages. Seven days after inoculation with *P. myriotylum*, the plants were scored for 5 days with the following scale: 0 = plants remain green and healthy; 1 = leaf sheath collar discolored and lower leaves turned yellow; 2 = plants alive, but shoots either totally yellow or dead; and 3 = all shoots dead (Stirling et al., 2009). The disease index (DI) was calculated using the equation DI = Σ 18_*i*=1_ Xi/54 where DI is the disease index rating from 0 (healthy) to 1 (dead), Xi is the disease rating of the ith replicate (from 1 to 18), and 54 is equal to the number of replicates multiplied by the highest rating scale of 3. The disease control rate was calculated by the following equation: (“*P. myriotylum* inoculated plants” - “*P. oligandrum* and *P. myriotylum* inoculated plants”)_*diseaseindex*_/*P. myriotylum*_*diseaseindex*_ × 100%.

To demonstrate that *P. oligandrum* could be recovered from around the roots of plants where there was a biocontrol effect on the PSR, the vermiculite from around the roots was collected into water containing the following five antimicrobial compounds: 100 μg/mL ampicillin, 50 μg/mL nystatin, 10 μg/mL pentachloronitrobenzene, 50 μg/mL rifampicin and 100 μg/mL carbenicillin. Serial dilutions of the suspension were plated onto 10% V8 agar containing the same antibiotics as above, and isolated colonies were sub-cultured onto V8 agar for DNA extraction using a CTAB-based extraction method. For PCR from the gDNA samples, ITS1 and ITS4 primers (White et al., 1990) were used to identify the species present. The 25 μL PCR reactions consisted of a 2X Taq Master Mix (VAZyme), 0.4 μM of each primer and 2 μL of gDNA template or water. The PCR cycling conditions were 95°C for 3 min, followed by 35 cycles of 95°C for 15 s, 56°C for 15 s, 72°C for 1 min, and then 72°C for 5 min. After agarose gel analysis, PCR products from the reactions using the ITS1 and ITS4 primers were sequenced using Sanger sequencing (Sangon Biotech).

## Results and Discussion

### The GAQ1 Isolate Belongs to *P. oligandrum*

The *P. oligandrum* isolate GAQ1 was recovered from soil from a field where infected ginger with symptoms of PSR disease was growing. The ITS region sequence of GAQ1 (MK774755.1) is 99.87% similar (788/789) to the strain of *P. oligandrum* CBS 382.34 (AY598618.2) used in the taxonomic monograph of the *Pythium* genus of van der Plaats-Niterink (1981). For three other molecular marker sequences compared to the CBS 382.34 strain, the GAQ1 isolate was 99.69% similar to the *CoxI* sequence, 99.81% similar to the *CoxII*, and 99.87% similar to the β*-tubulin* sequence. In a multi-locus phylogenetic analysis (using the ITS region, *CoxI*, *CoxII* and β*-tubulin* sequences), GAQ1 formed a clade with the *P. oligandrum* CBS 382.34 strain and with genome-sequenced *P. oligandrum* strains (Supplementary Figure 2). Previous reports of the ability of *P. oligandrum* to control other diseases caused by *Pythium* spp. such as *P. ultimum*, e.g., Abdelzaher et al. (1997), prompted an investigation into whether the newly recovered isolate of *P. oligandrum* could antagonize *P. myriotylum*, and what transcriptional changes occurred during their interaction, and whether *P. oligandrum* could control PSR of ginger caused by *P. myriotylum*.

### Visualization of *P. oligandrum* and *P. myriotylum* Interaction

**FIGURE 1:**
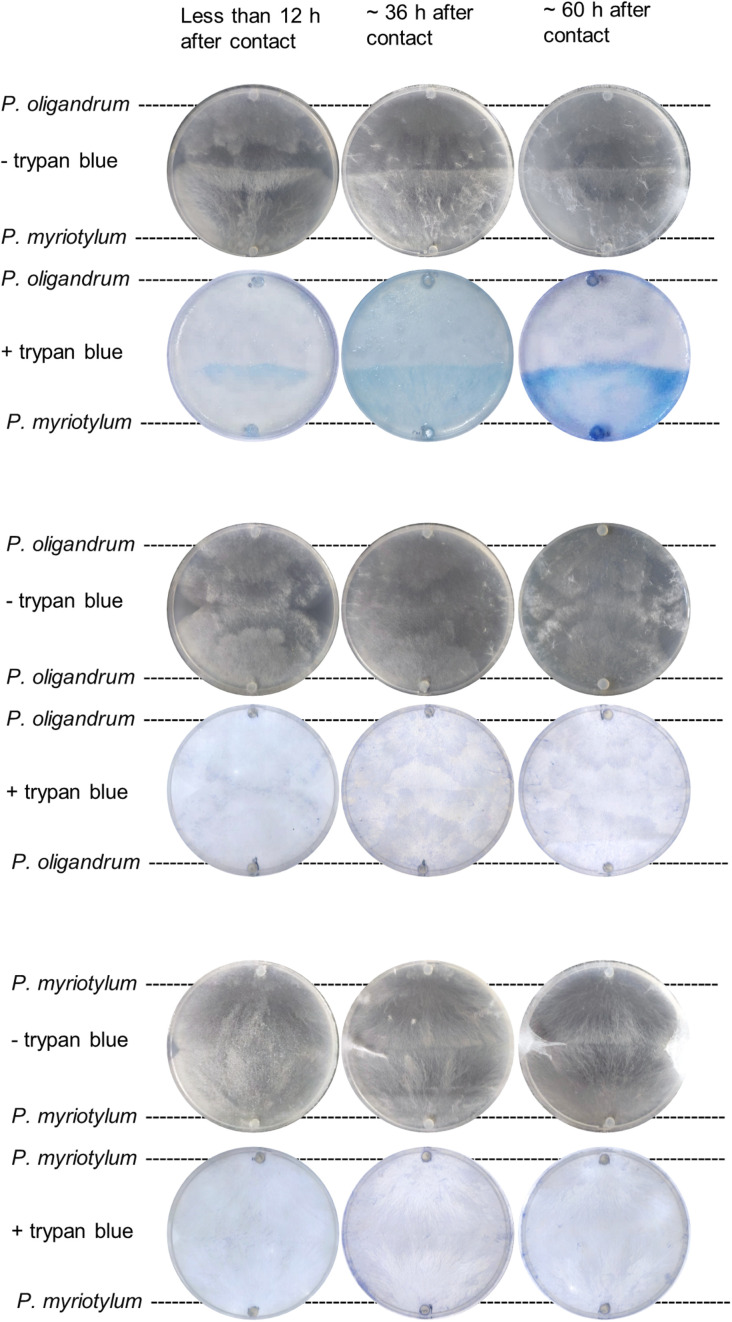
Representative images of trypan blue staining of mycelia from *P. myriotylum* and *P. oligandrum* self- or mixed-species confrontation demonstrating that *P. myriotylum* mycelium becomes non-viable in the presence of *P. oligandrum*. Images before and after trypan blue staining of the cultures are shown from less than 12 h, from ∼ 36 h and from ∼60 h after contact of the hyphal fronts of the colonies. The brightness and contrast of the images was adjusted consistently. The images from the control self-confrontation cultures demonstrate that the blue-colored staining does not appear in the self-confrontation.

We were interested in understanding if the *P. oligandrum* GAQ1 isolated from soil where infected ginger was growing could control diseases of ginger such as *Pythium* soft rot. Previously, we recovered a virulent isolate of *P. myriotylum* named SWQ7 that could infect and kill ginger plants (Daly et al., 2021). To understand whether the GAQ1 isolate could antagonize *P. myriotylum*, we used plate-based confrontation assays and microscopy coupled with vitality staining using trypan blue. Both *P. oligandrum* and *P. myriotylum* had similar growth rates on V8 medium. Before contact of the mycelial fronts of both species, blue staining with trypan blue (indicating the presence of non-viable mycelium) was not observed from either species. Several hours after contact, the confrontation zone showed staining with the trypan blue (Figure 1), indicating a loss of viability in some of the hyphae. As the incubation of other cultures were continued for longer periods, the blue-stained area was seen to expand to cover the entire side of the plate where *P. myriotylum* had previously been growing on. The pattern of the trypan blue staining suggested successful antagonism by *P. oligandrum* of *P. myriotylum* as the loss of viability of the hyphae only appeared to occur in the part of the cultures where *P. myriotylum* was growing before the confrontation with *P. oligandrum*. Of note, when *P. oligandrum* or *P. myriotylum* was confronted with itself, the occurrence of trypan blue staining was not observed (Figure 1).

**FIGURE 2:**
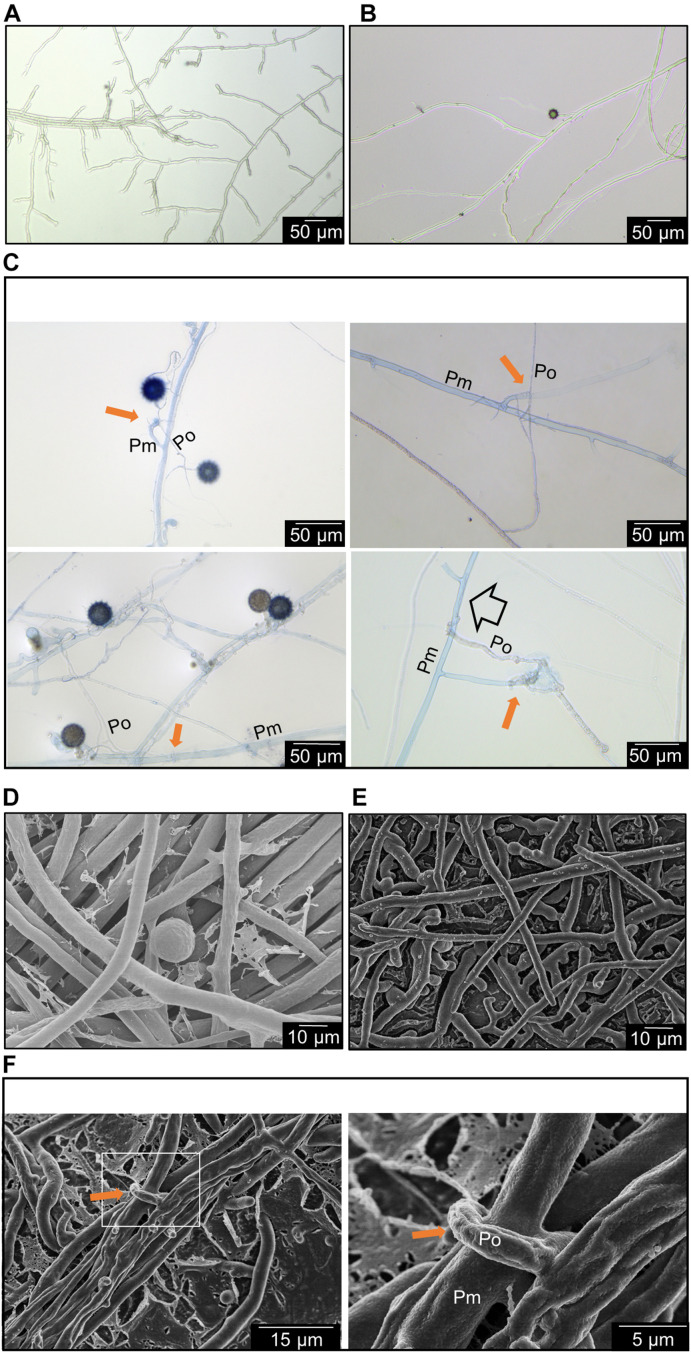
Representative features of the interaction between *P. myriotylum* (Pm) *and P. oligandrum* (Po). Here hyphae are imaged in the region where the two colonies have recently made contact using either brightfield microscopy coupled with trypan blue staining **(A–C)** or cryo-scanning electron microscopy **(D–F)**. **(A)** *P. myriotylum* from self-confrontation, **(B)** *P. oligandrum* from self-confrontation with spiny oogonia visible, **(C)** coiling of *P. oligandrum* hypha around the hypha of *P. myriotylum* and apparent penetration by *P. oligandrum* of *P. myriotylum* hypha. The brightfield images of the cultures from where *P. oligandrum* and *P. myriotylum* confront each other were stained with trypan blue. **(D)** *P. myriotylum* from self-confrontation, **(E)** *P. oligandrum* from self-confrontation, **(F)** coiling of a *P. oligandrum* hypha around a hypha of *P. myriotylum* at two different magnifications. The cryo-SEM images were taken from cultures growing on polycarbonate membranes using the same experimental set-up as was used for the Dual-RNAseq (see Supplementary Figure 1 for images of these cultures). The solid amber arrow indicates examples of hyphal coiling and the empty arrow indicates an example of penetration.

The brightfield microscopy revealed that the trypan blue staining occurred on the thicker hyphae which are generally found from *P. myriotylum* while the thinner hyphae of *P. oligandrum* lack the trypan blue stain. Figures 2A,B demonstrate that the size of *P. myriotylum* hyphae is larger than those of *P. oligandrum*. Clear occurrences of hyphal coiling were observed where the thinner hyphae of *P. oligandrum* coiled around the thicker trypan blue-stained hyphae of *P. myriotylum*, with also occurrences of penetration of the *P. myriotylum* hyphae by *P. oligandrum* (Figure 2C). Cryo-SEM was used to obtain higher magnification and resolution images of the hyphal coiling (Figures 2D–F). In the brightfield images, there were also occurrences of the spiny oogonia on the part of the plate where *P. myriotylum* had previously been growing. Spiny oogonia are one of the characteristic features of *P. oligandrum* and related species (van der Plaats-Niterink, 1981) and are not formed by *P. myriotylum*. The observation here of the spiny oogonia further demonstrates the colonization by *P. oligandrum* of the part of the plate where *P. myriotylum* had previously been growing.

The analysis here suggests a clear parasitic interaction of the *P. oligandrum* GAQ1 isolate on *P. myriotylum*. The macroscopic observations of the loss of vitality of the *P. myriotylum* hyphae after contact with *P. oligandrum* coupled with the microscopic observations of hyphal coiling support this parasitic mechanism. Previously, there are reports of the *P. oligandrum* isolate CGH1 antagonizing multiple *Pythium* spp. with observances of hyphal coiling and hyphal penetration (Berry et al., 1993). Interestingly, in the study of Berry et al. (1993), a lack of antagonism from the *P. oligandrum* isolate toward the plant pathogen *P. aphanidermatum* was found, and instead *P. aphanidermatum* coiled around the hyphae of *P. oligandrum*. The two species of *P. aphanidermatum* and *P. myriotylum* are relatively closely related, being from the *Pythium* clades A and B, respectively (Lévesque and De Cock, 2004), but in our study, we did not find occurrences of *P. myriotylum* hyphae coiling around the hyphae of the *P. oligandrum* GAQ1 isolate.

### Overview of Transcriptome Analysis of the Interaction Between *P. oligandrum* and *P. myriotylum*

**FIGURE 3:**
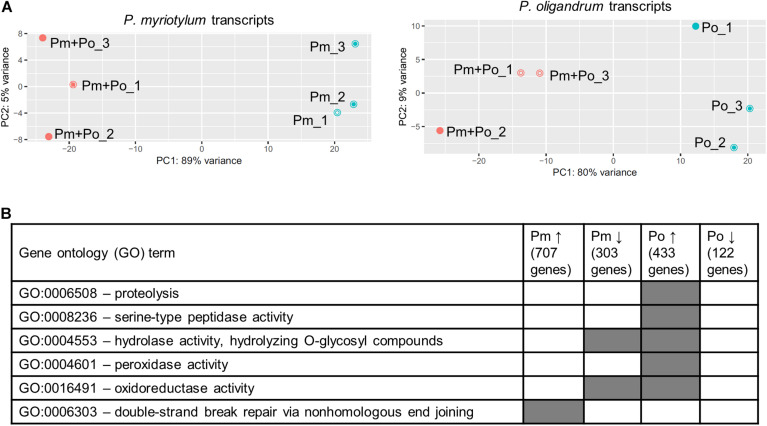
**(A)** Overview of the clustering pattern from principal component (PC) analysis of the transcripts from the replicate cultures from *P. oligandrum* (Po) and *P. myriotylum* (Pm) self- or mixed-species confrontations, **(B)** summary of interesting gene ontology (GO) terms enriched in the differentially expressed genes in *P. myriotylum* and *P. oligandrum* when they confront each other compared to a self-confrontation. The full list of enriched GO terms can be found in Supplementary Table 4.

The interaction between *P. oligandrum* and *P. myriotylum* as observed macro- and microscopically was used as the basis to sample mycelia for transcriptomic analysis when *P. myriotylum* and *P. oligandrum* confronted each other or themselves. The part of the mycelia indicated by the black dotted-line in Supplementary Figure 1. were sampled for RNAseq which corresponds to an early time-point in the interaction between *P. oligandrum* and *P. myriotylum*. The *P. oligandrum* GAQ1 isolate had a flatter colony morphology with a notable chrysanthemal pattern to the mycelia, compared to the *P. myriotylum* SWQ7 isolate, which had a fluffier colony appearance with considerable numbers of aerial hyphae (Supplementary Figure 1). Approximately 20 million reads were obtained from each sample with ∼ 90% of the reads aligning to one location in a concatenation of the genome assemblies of the two species (Supplementary Table 3). When the reads that aligned uniquely to gene models from each species from the mixed species confrontation samples were counted, similar numbers of reads were counted on gene models for each species, with the biggest difference being only twofold in replicate two (Supplementary Table 3). From the single-species cultures of *P. oligandrum* or *P. myriotylum*, very few reads were counted on the gene models from the species not found in the culture, i.e., at between 0.005 and 0.06% of the reads that were counted on the gene models of the species present in the single culture (Supplementary Table 3). The above analysis indicated that the genes from the two species are sufficiently different in sequence to be distinguished from each other. The clustering pattern from a principal component analysis of the expression levels of the genes from each species showed separate clustering of the replicates from the self-confrontation compared to when the two species confronted each other (Figure 3A). In terms of the number of differentially expressed genes, there were 707 genes upregulated and 315 genes downregulated in *P. myriotylum* in the interaction with *P. oligandrum*. In *P. oligandrum*, there were 433 genes upregulated and 122 genes downregulated in the interaction with *P. myriotylum* (Figure 3B and Supplementary Table 1).

### Transcriptomic Aspects of Parasitic Mechanisms From *P. oligandrum*

Cellulose is a key component of the oomycete cell wall (Mélida et al., 2013), and a potential major target of any antagonist of an oomycete like *P. myriotylum*. Based on CAZy, signal peptide and transmembrane domain annotations, *P. oligandrum* has 50 putative host/prey targeted cellulases. Host/prey targeted cellulases were considered those cellulases which contained a signal peptide and lacked transmembrane domains indicating that they are likely secreted into the extracellular environment (where they could degrade cellulose found in the cell walls of both a plant and oomycete host) and are unlikely to be involved in remodeling of the *P. oligandrum* cell wall. These host/prey targeted cellulases were from CAZy families GH1, GH3, GH5, GH6, GH7, GH30_1, GH131 and AA9. Of the putative host/prey targeted cellulases, 11/50 were significantly upregulated in the interaction with *P. myriotylum*, and notably, none of these 50 was significantly downregulated (Supplementary Table 1C). In general, the *P. oligandrum* putative host/prey targeted cellulases were expressed but were not upregulated to a high level in the presence of *P. myriotylum*. Only two of the upregulated host/prey targeted cellulases had an FPKM > 100 (two GH5_14 members that likely encode for β-glucosidase activity). Two members of the GH30_1 sub-family that likely also encode for β-glucosidase were constitutively expressed with FPKM > 100 but there were no good examples of upregulated host/prey targeted cellulases that likely have activity on the cellulose polymer (e.g., cellobiohydrolases or endoglucanases). *P. oligandrum* protease or peptidase encoding genes were a major category of genes that were upregulated in the interaction with *P. myriotylum* e.g., the GO term for—proteolysis (GO:0006508) was enriched in the *P. oligandrum* genes upregulated in the interaction with *P. myriotylum* (Figure 3B). The putative role of *P. oligandrum* proteases will be described in more detail in a later section.

The limited upregulation of the host/prey targeted cellulases in *P. oligandrum* in the confrontation with *P. myriotylum* contrasts somewhat with their expression in *P. oligandrum* in the interaction with *P. infestans* from the study of Liang et al. (2020). Here the majority of the AA9 cellulases were upregulated (with a subset expressed at a high level) at 12 and 24 h after contact between *P. oligandrum* and *P. infestans* compared to control samples before contact was made (Liang et al., 2020). Differences in the timing of the sampling for expression analysis, using a different *P. oligandrum* strain (CBS530.74), or other hosts (e.g., differences in the cell wall composition between the hosts) could contribute to the differences in cellulase gene expression when *P. oligandrum* confronted *P. myriotylum* compared to when *P. infestans* was confronted. The cellulolytic and protease gene expression measured from the GAQ1 isolate likely contributes to the effects seen in the microscopy images whereby there are clear signs of a loss of viability of the *P. myriotylum* mycelium from the trypan blue staining (Figures 1, 2) and examples of hyphal penetration by *P. oligandrum* of the *P. myriotylum* hyphae (Figure 2C).

Apart from genes involved in a parasitic mechanism, several known genes from *P. oligandrum* with a role in mediating the biocontrol of diseases via stimulation of plant defenses, were highly expressed at a constitutive level. e.g., oligandrin *Po_g9166.t1* (*Oli−D1*) and *Po_g9168.t1* (*POD-2*) (there is no evidence that the presence of *P. myriotylum* downregulated these genes). Oli-D1 and POD-2 are elicitin/elicitin-like proteins, and seven other *P. oligandrum* elicitin-like genes were upregulated in the confrontation with *P. myriotylum*, but their expression level was 10–100-fold less than *Oli-D1* and *POD-2* (Supplementary Table 1).

### Features of the Transcriptomic Response of *P. myriotylum* When Parasitized by *P. oligandrum*

**FIGURE 4:**
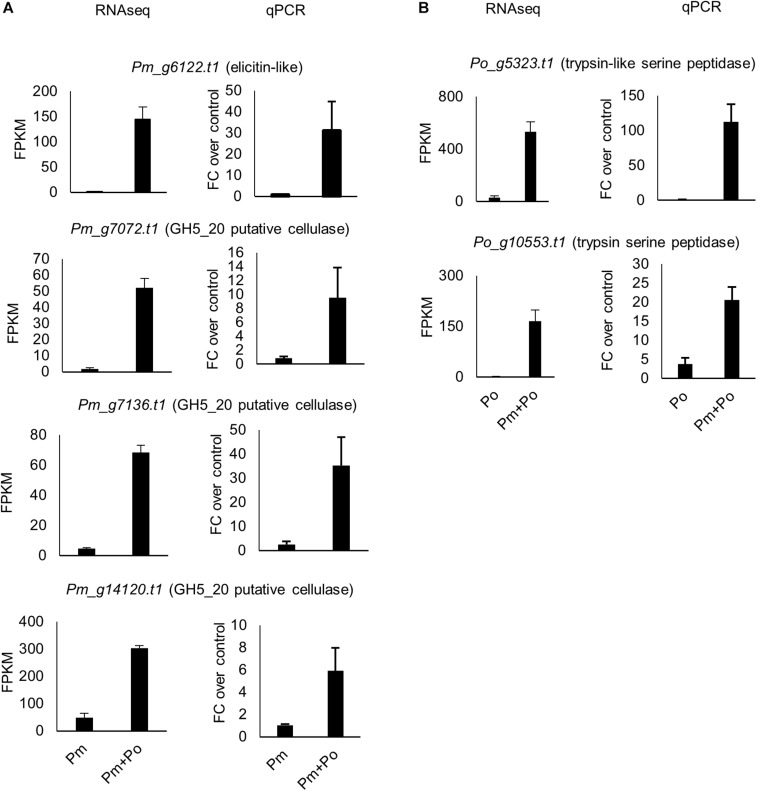
Expression levels from RNAseq and qPCR for selected **(A)** *P. myriotylum* genes and **(B)** *P. oligandrum* genes upregulated in the mixed-species confrontation compared to the self-confrontation. The error bars represent standard errors (*n* = 3). The expression levels of *P. myriotylum* were normalized using the two reference genes *Pm_g6466.t1* (tubulin) and *Pm_17033.t1* (gapdh) and the expression levels of *P. oligandrum* were normalized using *Po_g2472.t1* (succinate dehydrogenase) and *Po_g6912.t1* (g6pdh). FC, fold change; Pm, *P. myriotylum*; Po, *P. oligandrum*.

A subset of *P. myriotylum* putative cellulase genes were upregulated in the confrontation with *P. oligandrum*. In contrast, to the *P. oligandrum* upregulated cellulases described in the previous section, a smaller number of *P. myriotylum* cellulases were upregulated, and at least half seemed likely to have a cellulose remodeling or intracellular role rather than as part of a counter-attacking defensive strategy. Six *P. myriotylum* genes annotated as putative cellulases were upregulated: *Pm_g1192.t1* (GH5_14), *Pm_g1197.t1* (GH5_14), *Pm_g7072.t1* (GH5_20), *Pm_g7136.t1* (GH5_20), *Pm_g7449.t1* (AA9), and *Pm_g14120.t1* (GH5_20). Two of the putative cellulases were annotated with a signal peptide (*Pm_g1192.t1* and *Pm_g7449.t1*) and three of those without a signal peptide were annotated with transmembrane domains (*Pm_g7072.t1*, *Pm_g7136.t1*, and *Pm_g14120.t1*), suggesting these three are membrane-bound proteins. The six upregulated *P. myriotylum* putative cellulases were annotated as either GH5_14, GH5_20, or AA9 family members. The characterized activities within the GH5_14 family include β-glucosidase and exo-β-1,3-glucosidase activities.^2^ The β-glucosidase activity could act on the cellobiose released from cellulose but cellulose does not contain β-1,3 linked glucans. The GH5_20 members appear to only be found in Stramenopiles and appear most closely related to GH5_1 members, of which many have been characterized as endo-β-1,4-glucanases (Aspeborg et al., 2012) but no GH5_20 members appear to have been characterized to date. The expression levels of the three putative cellulases from the GH5_20 family encoded by *Pm_g7072.t1*, *Pm_g7136.t1*, and *Pm_g14120.t1* were validated by qPCR (Figure 4A). For the other CAZy family found amongst the upregulated cellulases; generally, proteins containing an AA9 domain have oxidative activity toward crystalline cellulose (Quinlan et al., 2011). The putative transmembrane domain-containing cellulases upregulated in *P. myriotylum* when confronted with *P. oligandrum* may remodel the cellulose in the *P. myriotylum* cell wall as part of a defensive response to the *P. oligandrum* antagonism. In mycoparasitic interactions involving *Trichoderma atroviride*, the remodeling of prominent fungal cell wall polysaccharides chitin and chitosan was important for the mycoparasitic ability of the fungus by contributing to self-defense (Kappel et al., 2020).

A subset of nine *P. myriotylum* elicitin-like putative pathogen-associated molecular pathogen (PAMP) molecules were upregulated in the confrontation with *P. oligandrum*. The upregulation of the elicitin-like *Pm_g6122.t1* was validated by qPCR (Figure 4A). Of note, other elicitin-like proteins are highly constitutively expressed, such as *Pm_g10365.t1* with an FPKM ∼ 20,000 that are more likely to function in *P. myriotylum* in the major primary role of the elicitin-like proteins in scavenging for sterols (Janků et al., 2020). If the upregulation of a subset of the *P. myriotylum* elicitin-likes found in the *in vitro* confrontation was replicated in the tri-partite interaction of the two *Pythium* spp. and a plant host of *P. myriotylum* (e.g., ginger), there is the potential to modulate plant immunity and the virulence of *P. myriotylum*, as elicitin-like proteins from other oomycete species can function as PAMPs (Derevnina et al., 2016). This, of course, would be an unintentional consequence of the interaction of *P. oligandrum* on *P. myriotylum* as a bioeffector does not intentionally alter the virulence of the plant pathogen it antagonizes as instead, the plant pathogen is a nutrient source or a competitor for ecological resources.

The GO term for double-strand break repair via non-homologous end-joining (GO:0006303) was enriched in the genes upregulated in *P. myriotylum* in the interaction with *P. oligandrum* (Supplementary Table 4). The two genes responsible for this enrichment, *Pm_g12183.t1* and *Pm_g10950.t1*, were annotated with domains found in Ku70/Ku80 proteins (PF02735, PF03730 and PF03731). Ku proteins are evolutionarily conserved proteins that mediate repair of double-stranded DNA by non-homologous end-joining of the broken ends of the DNA (Doherty and Jackson, 2001). Although these two genes were also expressed in *P. myriotylum* when it confronts itself, their upregulation in the confrontation with *P. oligandrum* suggests there could be a greater requirement to repair double-strand breaks.

In *P. myriotylum*, there were relatively few protease-encoding genes upregulated in the confrontation with *P. oligandrum*. Instead, one stand-out part of the interaction between *P. myriotylum* and *P. oligandrum* was the high upregulation of *P. myriotylum* putative protease inhibitors along with the upregulation or constitutive expression of *P. oligandrum* proteases, which will be described in detail in the next section.

### High Upregulation of a Sub-Set of *P. myriotylum* Kazal-Type Protease Inhibitors and *P. oligandrum* Peptidases

**FIGURE 5:**
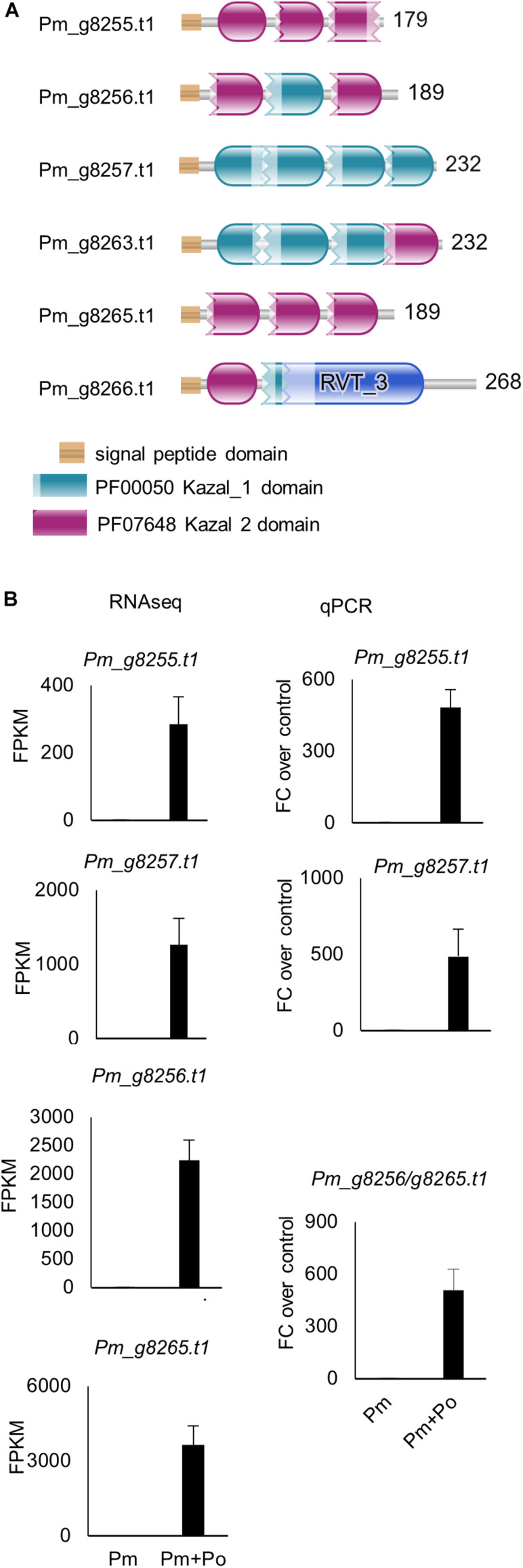
**(A)** Protein sequences of the confrontation-upregulated *P. myriotylum* Kazal-type protease inhibitors highlighting the locations of the Kazal domains. **(B)** Expression levels from RNAseq and qPCR of a sub-set of the *P. myriotylum* Kazal-type protease inhibitors upregulated in the confrontation with *P. oligandrum* compared to the self-confrontation. The error bars represent standard errors (*n* = 3). The expression levels of *P. myriotylum* were normalized using two reference genes *Pm_g6466.t1* (tubulin) and *Pm_17033.t1* (gapdh). Pm, *P. myriotylum*; Po, *P. oligandrum*.

In the *P. myriotylum* genome, there are 23 genes annotated with a Kazal-type protease inhibitor domain (Supplementary Table 1 and Figure 5A). One of the most striking features of the genes induced in the interaction between *P. myriotylum* and *P. oligandrum* was the upregulation of six of the *P. myriotylum* genes annotated with a Kazal-type protease inhibitor domain in the interaction with *P. oligandrum* (*Pm_g8255.t1*, *Pm_g8256.t1*, *Pm_g8257.t1*, *Pm_g8263.t1*, *Pm_g8265.t1*, and *Pm_g8266.t1*). Three of these genes (*Pm_g8256.t1*, *Pm_g8257.t1*, and *Pm_g8265.t1*) were induced greater than 1,000-fold to a high FPKM value of greater than 1000 FPKM. All six of these Kazal-type protease inhibitor genes were annotated with a signal peptide, and only *Pm_g8257.t1* is predicted to possesses a transmembrane domain, indicating that the other five are likely secreted into the environment around *P. myriotylum*. The induction of the genes annotated with a Kazal-type protease inhibitor domain was validated by qPCR (Figure 5B). Of the other Kazal-type protease inhibitor genes that were not upregulated, one of the genes (*Pm_g16340.t1*) appeared to be constitutively expressed when *P. myriotylum* was confronted by itself or by *P. oligandrum*, and two other Kazal-type protease inhibitor genes (*Pm_g8268.t1* and *Pm_g1759.t1*) were downregulated when *P. myriotylum* was confronted by *P. oligandrum*. Overall, for the *P. myriotylum* genes annotated with a Kazal-type protease inhibitor domain, the dominant feature was high upregulation of a subset of these when *P. myriotylum* was confronted by *P. oligandrum*. Of the genes upregulated in *P. myriotylum* in the interaction with *P. oligandrum*, the largest fold-change upregulated and highest expression was seen for these genes annotated as Kazal-type protease inhibitors.

In contrast to the *P. myriotylum* Kazal-type protease inhibitor genes, in *P. oligandrum*, none of the 13 genes annotated with a Kazal domain were differentially expressed, while one gene annotated with a Kazal domain (*Po_g7532.t1*) appeared to be constitutively expressed to a relatively high level of ∼500 FPKM. This gene, *Po_g7532.t1*, is a match to two EST sequences (EV245133.1 and EV243682.1) from an EST library sequenced from an *in vitro* interaction between *P. oligandrum* with heat-killed *P. infestans* mycelia (Horner et al., 2012). Another EST from the same library EV245779.1, annotated with a Kazal domain, matched *Po_g10361.t1*, which was expressed constitutively in the conditions at ∼70 FPKM.

Kazal-type protease inhibitors can inhibit serine proteases (Tian and Kamoun, 2005) and whether there was any upregulation of the class of proteases in *P. oligandrum* that the *P. myriotylum* Kazal-type protease inhibitors could inhibit was of interest. Interestingly, the GO enrichment analysis showed that the GO term for serine-type peptidase activity (GO:0008236) was enriched in the *P. oligandrum* genes upregulated in the confrontation with *P. myriotylum* (Figure 3B). The expression level of *P. oligandrum* putative serine proteases was assessed using the following Pfam annotations: Pfam00089 (Trypsin), Pfam13365 (Trypsin-like peptidase domain), Pfam00082 (Subtilase family), Pfam00450 (Serine carboxypeptidase), and Pfam05577 (Serine carboxypeptidase S28). Of these 142 *P. oligandrum* serine proteases, there were 23 upregulated when *P. oligandrum* confronted *P. myriotylum* compared to when *P. oligandrum* confronted itself. All of these 23 putative serine proteases were annotated with a signal peptide sequence (Supplementary Table 1). The upregulation of the peptidase with one of the largest fold changes in upregulation (*Po_g10553.t1*, a trypsin family serine protease) and the peptidase that had the highest expression level in the confrontation with *P. myriotylum* (*Po_g5323.t1*, a trypsin-like serine protease) was validated by qPCR (Figure 4B).

There is potential for these proteins with Kazal-type protease inhibitor domains to be involved in inhibition of the serine peptidases upregulated or constitutively expressed in *P. oligandrum*. Previously in the interaction between the biocontrol rhizobacterium *L. capsici* and the pathogen *P. infestans* (Tomada et al., 2017) there was an upregulation of a subset of Kazal-type protease inhibitors, and it was hypothesized that these could be involved in inhibiting lytic activities from *L. capsici*. Although, notably, Tomada et al. (2017) did not consider overall an active response from *P. infestans* to the antagonism from *L. capsici*. Consistent with a prominent role for *P. oligandrum* proteases in the antagonism of *P. myriotylum* was a lack of upregulation of the *P. oligandrum* Kazal-type protease inhibitors, as their upregulation could inhibit the *P. oligandrum* protease activity. Horner et al. (2012) considered the ESTs annotated with a Kazal domain as part of a defense or counter-defensive response of *P. oligandrum* to heat-killed *P. infestans* mycelia. However, in the confrontation between *P. oligandrum* and the living *P. myriotylum* mycelia, the *P. oligandrum* putative Kazal-type inhibitors appear to play a minor role.

While the transcriptomic response of *P. myriotylum* in the confrontation with *P. oligandrum* suggested aspects of a defensive or counter-attacking strategy, this appears insufficient to defend against *P. oligandrum* as clearly in the plate confrontation assays, *P. oligandrum* caused progressive loss of viability in the hyphae of *P. myriotylum* (Figure 1). As the ability to antagonize on plate cultures suggested it could have a control effect on diseases caused by *P. myriotylum*, the ability to control the Pythium soft-rot of ginger caused by *P. myriotylum* was investigated using a pot-trial experiment.

### *P. oligandrum* GAQ1 Has Control Effect on Pythium Soft-Rot of *P. myriotylum* Infected Ginger

**FIGURE 6:**
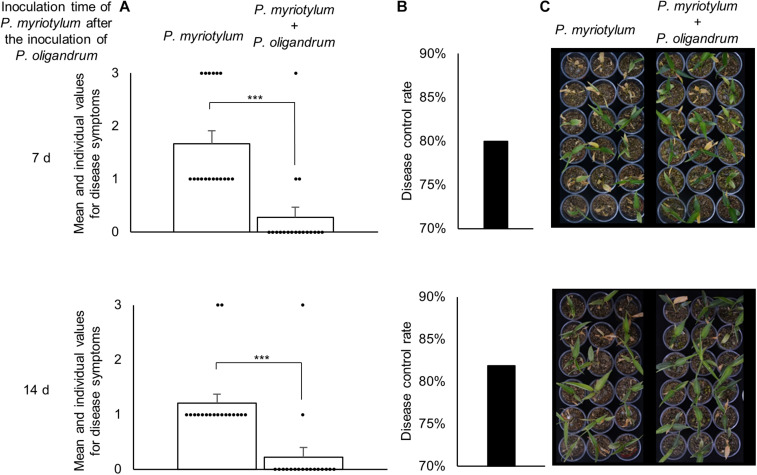
Biocontrol effect of *P. oligandrum* on Pythium soft-rot (PSR) of ginger disease caused by *P. myriotylum* from two different inoculation time-points of *P. myriotylum* after the inoculation of *P. oligandrum*. **(A)** The mean values and raw data from the scoring of the disease symptoms (scale from 0 to 3) in the 18 replicate plants for each condition. ***corresponds to a *P* ≤ 0.001 from a Mann-Whitney Rank Sum Test (*n* = 18). **(B)** The disease control rate of *P. oligandrum* on PSR of ginger. **(C)** Images of the 18 replicate plants inoculated with *P. myriotylum or P. myriotylum* and *P. oligandrum*. The data and images were recorded 11 d after the inoculation of the plants with *P. myriotylum*. The scoring for the disease symptoms was always zero for the plants that were inoculated with only *P. oligandrum*, and for the plants that were not inoculated with either of the *Pythium* spp.

As *P. oligandrum* demonstrated an ability to antagonize *P. myriotylum* in plate confrontation assays, its ability to control PSR of ginger was investigated. *P. oligandrum* was inoculated first followed by inoculations of *P. myriotylum* at two time points after the inoculation of *P. oligandrum*, and this also led to inoculations of *P. myriotylum* onto plants of two different ages and sizes. Clearly from the data on the above-ground disease symptoms in Figure 6, the plants inoculated with *P. myriotylum* developed the disease symptoms. The disease was more severe in the younger and smaller ginger plants as a higher proportion of the plants developed the more severe symptoms whereby all the aerial parts of the plant appeared dead (Figure 6A). In both of the time-points analyzed, the presence of *P. oligandrum* resulted in a clear and significant (*P* < 0.001) reduction in the disease symptoms whereby > 80% of the plants had no disease symptoms (Figure 6A). The disease control rate was ∼80% in both inoculation time points (Figure 6B). To show that *P. oligandrum* could be recovered from plants where there was a biocontrol effect on the PSR, *P. oligandrum* was re-isolated from around the roots that had been infected with *P. myriotylum* (Supplementary Figure 3).

A similar experiment using a different *P. oligandrum* isolate to control PSR soft-rot of ginger caused by a *P. myriotylum* isolated from infected ginger in Australia did not reduce the disease symptoms on ginger plants (Le, 2016). It was speculated by Le (2016) that the lack of a control effect may have been in part due to poor colonization of the soil by the *P. oligandrum* isolate (vermiculite was used to grow the ginger plants inoculated with GAQ1) or the ineffective application of the oospore suspension by soil drenching (mycelia of the GAQ1 isolate was added directly into the vermiculite). However, there is support for *P. oligandrum* to control other diseases caused by *P. myriotylum* where previously it was shown that *P. oligandrum* was able to reduce soybean damping-off caused by *P. myriotylum* (You et al., 2019).

Part of the rationale for the suite of analyses undertaken in this study involving microscopy, dual-transcriptomics, and biocontrol was to look for further insights into why biocontrol strategies in the field are often hampered by a lack of robustness or durability. Of course, dominant reasons for this include interactions between the bioeffector and a complex microbiome, varying abilities of the bioeffector to propagate, and changes in host strain or isolate (i.e., the plant pathogen) from year to year. The biocontrol effect from the controlled laboratory conditions is promising, with a disease control rate of ∼80% for the application of *P. oligandrum* to control PSR of ginger, although the ability of *P. oligandrum* to suppress PSR of ginger in field conditions has yet to be tested. Suppose one were to extrapolate the microscopic and transcriptomic observations from the *in vitro* confrontation assays to the tri-partite interaction in the pot trial experiment. In that case, there is likely *P. oligandrum* parasitism or predation of *P. myriotylum* contributing to the suppression of the disease. The defensive or counter-attacking strategies suggested from the dual-transcriptomics, such as the Kazal-type protease inhibitor upregulation, do not appear to manifest themselves into a successful defense. In the *in vitro* plate confrontation assays, *P. oligandrum* progressively parasitized *P. myriotylum* mycelia, and a relatively high control rate of ∼80% was maintained in the pot-trial experiment. However, these defensive or counter-attacking strategies suggested by the dual-transcriptomics are another factor that is worthwhile in considering for the robustness and durability of biocontrol strategies in the field.

## Conclusion

The *P. oligandrum* isolate GAQ1 is a promising bioeffector of PSR disease of ginger caused by *P. myriotylum* and has parasitic abilities that likely contribute to the control of PSR disease of ginger. Likely side-by-side comparisons of different *P. oligandrum* strains would be required to determine whether the GAQ1 isolate is superior for control of particular diseases (e.g., whether *P. oligandrum* GAQ1 which was isolated from a field with infested ginger is superior to other strains in controlling the PSR of ginger). For field-based application, an oospore-based preparation of *P. oligandrum* GAQ1 would likely ensure better shelf-life of the bioeffector compared to the use of a mycelial-based inoculum. The use of dual-transcriptomics is a powerful approach to uncover differentially expressed genes in the interaction between an antagonist and its prey or host. The differentially expressed genes such as Kazal-type protease inhibitors are promising candidates to investigate further molecular aspects of the antagonism and defensive responses between host and prey.

## Data Availability Statement

The datasets presented in this study can be found in online repositories. The names of the repository/repositories and accession number(s) can be found in the article/Supplementary Material.

## Author Contributions

PD, SC, QZ, JZ, JL, and TX performed the experiments. PD, DF, and JM performed bioinformatic analyses. PD and SC wrote the manuscript and analyzed the results. XW and XS sampled the soil for microbe isolation. LW obtained funding for the research. TS, DZ, SD, MJ, ID, and LW contributed to the discussion and manuscript revision. All authors contributed to the article and approved the submitted version.

## Conflict of Interest

LW, DZ, and QZ were co-authors on a patent application for *P. oligandrum* (Patent application number CN201910757035.2) relating to the biocontrol of plant diseases using the *P. oligandrum* GAQ1 isolate. The remaining authors declare that the research was conducted in the absence of any commercial or financial relationships that could be construed as a potential conflict of interest.

## Publisher’s Note

All claims expressed in this article are solely those of the authors and do not necessarily represent those of their affiliated organizations, or those of the publisher, the editors and the reviewers. Any product that may be evaluated in this article, or claim that may be made by its manufacturer, is not guaranteed or endorsed by the publisher.

## Footnotes

1
http://www-huber.embl.de/users/anders/HTSeq/doc/count.html

2
http://www.cazy.org/GH5_14_characterized.html
