# Supplementary material for: Dual-Transcriptomic, Microscopic, and Biocontrol Analyses of the Interaction Between the Bioeffector Pythium oligandrum and the Pythium Soft-Rot of Ginger Pathogen Pythium myriotylum
Source: Front Microbiol. 2021 Nov 16;12:765872. doi: 10.3389/fmicb.2021.765872 (PMC8637047; doi:10.3389/fmicb.2021.765872)
Supplement: Supplementary file 3 [file Table_3.PDF]

culture	replicate	total number of reads	% reads aligned once using HISAT2 aligner	HTSeq-count total of reads (unique) to *P. myriotylum* genes	HTSeq-count total of reads (unique) to *P. oligandrum* genes
P. myriotylum	1	15,496,493	87.27%	11,815,169	635
	2	19,252,066	86.97%	14,284,539	898
	3	22,957,591	87.51%	17,784,168	973
P. myriotylum and P. oligandrum	1	22,924,478	89.77%	8,827,290	9,629,565
	2	20,759,090	89.02%	11,295,982	5,109,743
	3	22,490,653	90.32%	8,779,361	9,810,633
P. oligandrum	1	22,300,672	91.89%	5,573	19,050,926
	2	21,978,319	92.33%	3,308	19,013,578
	3	23,271,113	91.62%	11,051	19,848,856

Table S3. Summary of the total number of reads from the RNAseq, percentage of reads that aligned once to the concatenated assembly of *P. myriotylum* and *P. oligandrum*, and total number of uniquely mapping reads counted to the *P. myriotylum* or *P. oligandrum* genes.
