# Supplementary material for: Dual-Transcriptomic, Microscopic, and Biocontrol Analyses of the Interaction Between the Bioeffector Pythium oligandrum and the Pythium Soft-Rot of Ginger Pathogen Pythium myriotylum
Source: Front Microbiol. 2021 Nov 16;12:765872. doi: 10.3389/fmicb.2021.765872 (PMC8637047; doi:10.3389/fmicb.2021.765872)
Supplement: Supplementary file 5 [file Image_1.pdf]

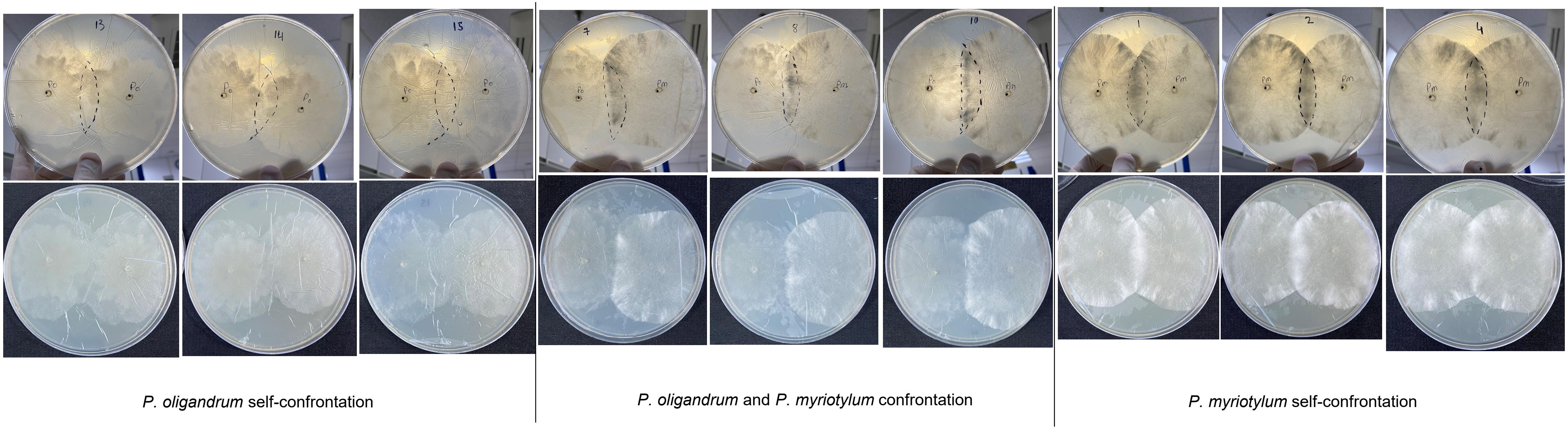

Supplementary Figure 1. Images of the single species and two species cultures of *P. oligandrum* and *P. myriotylum* at the time of sampling of RNA for transcriptomics analysis from each of the replicate plate cultures. The dotted-line indicates the part of the mycelia that was sampled.
