# Supplementary material for: Dual-Transcriptomic, Microscopic, and Biocontrol Analyses of the Interaction Between the Bioeffector Pythium oligandrum and the Pythium Soft-Rot of Ginger Pathogen Pythium myriotylum
Source: Front Microbiol. 2021 Nov 16;12:765872. doi: 10.3389/fmicb.2021.765872 (PMC8637047; doi:10.3389/fmicb.2021.765872)
Supplement: Supplementary file 6 [file Image_2.pdf]

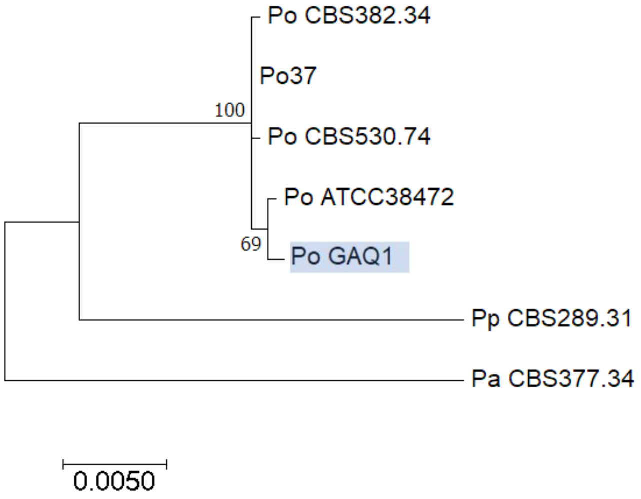

Supplementary Figure 2. Maximum likelihood tree using the concatenated alignments of the ITS region, *CoxI*, *CoxII* and *β-tubulin* sequences showing phylogenetic relationships among the *P. oligandrum* GAQ1 isolate from this work (highlighted), the *P. oligandrum* strain used in the description of van der Plaats-Niterink (1981) (CBS382.34) and three *P. oligandrum* strains which have had their genomes sequenced (Po37, ATCC38472 and CBS530.74). A *P. periplocum* (Pp) and a *P. acanthicum* (Pa) strain were used for outgroup sequences. There were a total of 2,509 positions in the final dataset. The numbers at the nodes represent the bootstrap values from a total of 100 replications. The scale bar represents 0.005 substitutions per site. The following sequence accessions or genome sequence locations were used. NAJK01000075.1:9386-10051 (CBS530.74 *β-tubulin*), MK774755.1 (GAQ1 ITS), MZ891585.1 (GAQ1 *CoxI*), MZ891586.1 (GAQ1 *CoxII*), MZ869812.1 (GAQ1 *β-tubulin*), SPLM01000150.1:45114-45837 (ATCC38472 ITS), SPLM01000017.1:24159-24806 (ATCC38472 *CoxI*), AF196611.1 (ATCC38472 *CoxII*), SPLM01000145.1:996514-997179 (ATCC38472 *β-tubulin*), AY598618.2 (CBS382.34 ITS), HQ708759.1 (CBS382.34 *CoxI*), KJ595381.1 (CBS382.34 *CoxII*), KJ595505.1 (CBS382.34 *β-tubulin*), LSAJ01000059.1:157656-158379 (Po37 ITS), LSAJ01000010.1:263793-264440 (Po37 *CoxI*), LSAJ01000493.1:22-543 (Po37 *CoxII*), LSAJ01000134.1:670-1335 (Po37 *β-tubulin*), AY598670.2 (CBS289.31 ITS), HQ708784.1 (CBS289.31 *CoxI*), KJ595369.1 (CBS289.31 *CoxII*), KJ595494.1 (CBS289.31 *β-tubulin*), HQ643409.1 (CBS377.34 ITS), HQ708456.1 (CBS37734 *CoxI*), KJ595380.1 (CBS377.34 *CoxII*), KJ595504.1 (CBS377.34 *β-tubulin*). Note that the CBS530.74 ITS, *CoxI* and *CoxII* sequences were obtained from sequencing PCR products from the CBS530.74 strain but these sequences were not deposited in GenBank.
