# Supplementary material for: Dual-Transcriptomic, Microscopic, and Biocontrol Analyses of the Interaction Between the Bioeffector Pythium oligandrum and the Pythium Soft-Rot of Ginger Pathogen Pythium myriotylum
Source: Front Microbiol. 2021 Nov 16;12:765872. doi: 10.3389/fmicb.2021.765872 (PMC8637047; doi:10.3389/fmicb.2021.765872)
Supplement: Supplementary file 7 [file Image_3.PDF]

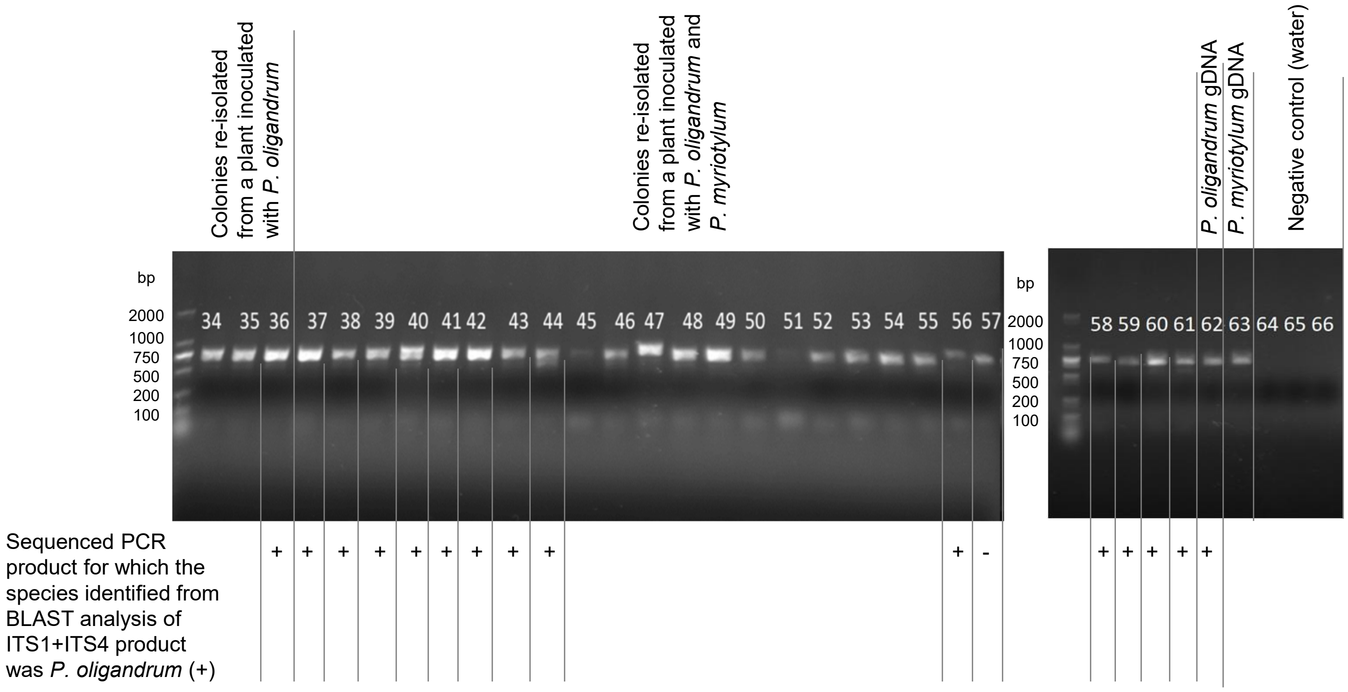

Supplementary Figure 3. Demonstration that *P. oligandrum* could be recovered from around the roots of plants where there was a biocontrol effect on the PSR. PCR reactions used the ITS1 and ITS4 primers with gDNA extracted from colonies isolated from the vermiculite surrounding the roots of ginger plants that were inoculated with either *P. oligandrum* or both *P. oligandrum* and *P. myriotylum*. A DL2000 marker was used to indicate the size of the PCR products. A sub-set of the PCR products were sequenced by Sanger sequencing and for these reactions, whether the species identified from BLAST analysis of the ITS1+ITS4 product was *P. oligandrum* is indicated. Note that samples 58-66 are from a separate agarose gel to samples 34-57.
